# The One Health Trail: Physician Leaders as Advocates for Human, Animal, and Environmental Health

**DOI:** 10.3389/fmed.2025.1735334

**Published:** 2026-01-12

**Authors:** Nikita Habermehl, Regan Denny, Sarah Fox, Chad Stickrath, Christina Reimer, Suzanne Brandenburg, Anuja Riles

**Affiliations:** 1 University of Colorado Anschutz School of Medicine at Colorado State University, Fort Collins, CO, United States; 2 University of Colorado Anschutz School of Medicine, Aurora, CO, United States

**Keywords:** advocacy, curriculum, leaders, medical students, One Health

## Abstract

The One Health Trail: Physician Leaders as Advocates for Human, Animal, and Environmental Health is a novel, nine-month longitudinal curriculum designed for third- and fourth-year medical students to explore the interconnectedness of human, animal, and environmental health through the One Health framework. This course integrates didactic, experiential, and independent learning activities, emphasizing real-world application through community-based engagement. Students interact with a diverse range of stakeholders. This includes but is not limited to: academic experts, researchers, policymakers, industry professionals, and community members. Areas of focus include the food supply chain, mining, wildlife and livestock management, water quality, human-animal bonds, ecosystem and planetary health, and healthcare sustainability. These interdisciplinary experiences cultivate students’ abilities to critically analyze complex health challenges and advocate for comprehensive, systems-level solutions. This manuscript outlines the course structure, details its educational components, and presents preliminary data from student course evaluations. Finally, the authors propose models for adapting this curriculum to other undergraduate medical education programs and to graduate-level training programs seeking to incorporate One Health principles into interdisciplinary learning.

## 1 Introduction

One Health is a collaborative and multidisciplinary approach to health care that acknowledges how the health of humans is intricately connected to the health of animals, agriculture, and our shared environment. It combines facets of climate change and planetary health with human and veterinary medicine, which is crucial to address health-related challenges within the intersection of these groups (1). At present, there are only a handful of undergraduate medical institutions across the United States that have integrated a One Health approach into their curriculum, educating professional students on these important relationships and encouraging interprofessional collaboration. While the Association of American Veterinary Colleges added One Health to the list of core competencies that all veterinary students should achieve in 2011, the U.S. medical education system is far behind (2). A survey of 133 U.S. medical schools in 2020 found that only 56% included One Health in the curriculum in some form, though the vast majority included in a single lecture or less (3). These authors were able to identify only seven medical institutions throughout the U.S. that offer a dedicated One Health elective or certificate course. This signals a major gap in medical education with an essential need for significant growth (4–6).

The concept of the intersection of human and animal health along with the public health lens is not a novel one and dates to the 19th century (7). This concept grew and developed over the next few decades to eventually be called “One Health.” It is more critical now than ever that medical schools educate students on the vital interconnectedness of human, animal, and environmental health. This is particularly important as the world becomes more connected, and the spread of zoonotic and water-borne diseases continues in a warming planet which leads to changes in animal and human health (8). At the University of Colorado School of Medicine at Colorado State University, the goal of the One Health curriculum is not only to introduce students to the concept, but to create student leaders with an expanded fund of knowledge who are equipped with the tools to incorporate One Health into their future practice and leadership roles as physicians. The course, entitled: One Health Trail: Physician Leaders as Advocates for Human, Animal, and Environmental Health covers an array of topics including but not limited to the modern food supply chain, mining, wildlife and livestock management, water safety and quality, human-animal bonds, planetary and ecosystem health, and healthcare sustainability. Key innovative components of this leadership course include (1) that it is championed by human physician faculty, (2) that in emphasizes collaboration with other healthcare and community professionals, including public health officials, veterinarians, and more, and (3) that it intentionally is grounded in the community in which the course is delivered. This manuscript will provide an overview of the structure of the novel course, discuss in detail the didactic, experiential, and independent learning activities that the students engage in, and subsequently present data to explore the impact of the course on our students. Lastly, the authors will discuss how this course could be translated to other medical education institutions and other graduate programs looking to expand their interdisciplinary curricular offerings in One Health.

## 2 Pedagogical framework and principles

The course One Health Trail: Physician Leaders as Advocates for Human, Animal, and Environmental Health was created using a well-structured pedagogical framework that emphasizes the interconnectedness of human, animal, and environmental health. Supported by key references from academic literature and organizational guidelines, the course directors relied on a synthesis of the following pedagogical frameworks and principles: constructivism, transformative learning theory, interdisciplinary and systems thinking, communities of practice, and experiential learning theory.

The constructivist learning approach is based on the principle that students learn best through active engagement and building upon prior knowledge and experiences (9, 10). The learners in this course were 3*^rd^* and 4*^th^* year medical students with a prior foundation in human health and certain One Health topics such as zoonotic disease and antimicrobial resistance. Yet, the students had limited formal engagement with many of the other domains of One Health. Constructivism was applied by utilizing case-based learning and real-world scenarios from our community to allow students to integrate existing knowledge with new environmental and veterinary health concepts. This often led to the use of transformative learning theory which underscores the concept that learning occurs through a shift in perspective, especially when engaging with complex, uncertain, or ethically challenging issues. Shifts in perspective were often noted as students built upon prior knowledge and preconceived notions about certain health challenges or the knowledge base of interdisciplinary experts.

One Health is unique in that it underscores the notion that health challenges are embedded in complex systems and that solutions to those challenges require multi-sectoral understanding and expertise (11). It is therefore essential to include an interdisciplinary and systems-thinking framework in any One Health curricular activity. Systems mapping, root cause analysis, and systems simulation are examples of pedagogical strategies used in the course to apply this framework.

The course directors further relied on the theory of experiential learning which emphasizes that learning is most effective when it involves concrete experience, reflection, conceptualization, and experimentation (12, 13). This was applied to the course by creating opportunities for students to experience One Health in action and to apply learning in new contexts through activities such as site visits and simulations.

Lastly, the course utilized the communities of practice framework which describes the concept that learning occurs through participation in the practices of community or that students learn by “doing” alongside experts (14). This was applied in the course through opportunities to work with professionals from veterinary, public health, and environmental sectors in joint activities. This interprofessional education further mirrors real-life One Health collaboration.

Taken together, the course blended the described pedagogical theories with the goals of promoting knowledge integration and conceptional learning, emphasizing interdisciplinary collaboration and systems literacy, and utilizing applied practice and real-world experiential learning opportunities.

### 2.1 Competency domains

Course directors utilized evidenced-based One Health Competencies and learning objectives that were previously described in the literature (11, 15–17). Table 1 demonstrates how the described pedagogical frameworks matched to the One Health competency domains to ensure that the competency domains aligned with the frameworks that were utilized in the course.

**TABLE 1:** Pedagogical framework and One Health competency domains.

Pedagogical framework	One Health competency domain	How it supports the competency
Constructivism	• One Health concepts and knowledge • Fundamentals of infectious disease	Promotes active building upon pre-existing knowledge of concepts in human health as well as construction of integrated knowledge across disciplines (human, animal, environment). Case-based learning supports conceptual transfer.
Experiential learning	• Collaboration and partnership • Ecosystem health • Infectious disease management	Provides real-world settings (e.g., fieldwork, simulations) where students engage with multisectoral professionals and complex environments.
Transformative learning	• Culture, Ethics, and values • Global health and social determinants	Utilizes disruptive experiences to encourage critical reflection on professional roles, ethical dilemmas, and equity in global health.
Situated learning/ communities of practice	• Collaboration and partnership • Policy, advocacy and regulation	Embeds students in interdisciplinary contexts, developing identity and skills through active participation with experts.
Systems thinking	• Collaboration, partnership and systems thinking • Epidemiology and risk analysis	Encourages analysis of complex interactions across human, animal, and environmental systems—key to addressing antimicrobial resistance, climate-related health threats.

## 3 Learning objectives, learning environment, and pedagogical format

### 3.1 Learning objectives

One Health competency domains were then used to create learning objectives for the course as demonstrated in Table 2. Short-term goals of the course include increased understanding of the One Health approach by students and sharing of clinical approaches used in human and veterinary medicine. Long-term outcomes are the broad integration of One Health in all fields, including human medicine. The projected impacts of the program are to assimilate the One Health approach into standard medical practice, to expand One Health knowledge, and to enhance health in all realms: human, animal, and environmental.

**TABLE 2:** One Health competency domains and One Health competencies.

One Health competency domain	Course learning objectives
One Health concepts and knowledge	Understand the conceptual definitions and scope of the One Health approach. Explain interconnections among human health, animal health, and environmental/ecosystem health. Describe interventions used to prevent disease and improve human, animal, plant, and environmental health at the individual, community, and population levels
Fundamentals of infectious diseases and infectious diseases management	Describe pathogens (viruses, bacteria, fungi, parasites) that have zoonotic potential, the mechanisms of spillover, incubation periods, and basic host-pathogen-environment interactions. Describe the main transmission routes for toxins, pathogens, and resistance genes, including human-animal-plant-environmental exposures, as well as vector-borne, waterborne, and airborne cycles. Participate in designing or planning outbreak responses; understand antimicrobial stewardship, diagnosis, treatment, and prevention in zoonotic infections.
Collaboration, partnership and systems thinking	Describe the relationship among various key One Health stakeholders locally and globally. Recognize the contributions of veterinarians, environmental scientists, and public health professionals in a collaborative health framework. Describe the benefits and challenges of a multidisciplinary integrative approach when implementing studies regarding health concerns at the human-animal-plant-environment interface
Ecosystem health	Analyze how air, water, food, and habitat influence human disease. Identify environmental risk factors for specific diseases. Recognize the impact of environmental changes (land use changes, climate change, biodiversity loss, etc.) on disease emergence; understand ecosystem services and health implications of ecosystem degradation.
Global health, social determinants, culture, ethics, and values	Identify common cultural and socioeconomic determinants and effects of illness, including poverty, residential geography, cultural practices, education, nutrition, and resource security Understand the effects of global change on health and how both local and global factors affect disease transmission within and between countries Recognize major challenges and opportunities to improve health in a global and local context through practical and applied training Compare and contrast health and non-health consequences of diseases and exposures, including social and behavioral, economic, and political effects across global regions
Policy, advocacy and regulation	Develop strategies to communicate the importance of One Health to patients, policymakers, and the public. Create effective advocacy messages tailored to different stakeholders Effectively communicate, both orally and in writing, scientific findings to the scientific community, non-health related academics, public audiences, media, and policy maker
Epidemiology and risk analysis	Explain epidemiologic principles used to characterize problems that involve human, animal, plant, and environmental components Understand scientific principles such as biological complexity, genetic diversity, and interactions of systems from individuals to ecosystems that influence modern complex challenges in human, animal, plant, and environmental health Explain how bio-surveillance, diagnostics, and therapeutic countermeasures are deployed

### 3.2 Learning environment

The One Health Trail was developed at a 4-year regional medical campus located at an institution of higher education that also has a Doctor of Veterinary Medicine (DVM) program. This leadership course is part of a larger longitudinal emphasis on One Health across our campus. This course is offered to 3*^rd^* and 4*^th^* year medical students and consists of a 2-week immersion, a 9-month independent, asynchronous learning period, and then another 2-week immersion. The course directors are two physicians with interests in One Health, advocacy, and curriculum development and delivery. They collaborated with interprofessional academic educators, community experts, public officials, non-profits, and private corporate partners with expertise in a variety of fields within and related to human, animal, and environmental health to serve as faculty that deliver the course content. Further details are described in the pedagogical format section below.

### 3.3 Pedagogical format

The course directors intentionally utilized a variety of pedagogical formats in the course, including lectures, small group discussions, case-based and peer-based learning, clinical integration, role-play and simulation, field visits, presentations, critical appraisal, and a comprehensive, culminating presentation. This was done to cater to diverse learning styles of students, increase engagement and motivation, and enhance knowledge retention through multiple methods of processing information. In addition, course directors aimed to harness the varied expertise, teaching styles, and engagement opportunities provided by faculty experts. Table 3 describes the immersion activities that took place in the two, two-week immersions. Table 4 describes the asynchronous activities that students engaged in during their 9-month independent, asynchronous block.

**TABLE 3:** Immersion learning activities.

Learning activity	Format	Led by
Introduction to One Health (1)	Didactic	Course director with background in One Health
Infectious diseases management (1)	Didactic, clinical integration	Centers for Disease Control and Prevention (CDC)– Fort Collins Campus
Science communication (1)	Didactic and small-group discussion	CDC - Fort Collins Campus
Ecosystem health, climate change, and environmental exposures (1)	Didactic	CDC – Fort Collins Campus
Water supply and safety (1)	Didactic and reflection	Community experts (non-profit and public sector)
Core One Health advocacy series (1, 2)	Didactic and small-group discussion	Course director with background in advocacy
Hospital sustainability (2)	Didactic	Local health system leader
Plague (1)	Case-based learning, clinical integration	Human medicine physician trained in infectious diseases
Tularemia (1)	Case-based learning, clinical integration	Human medicine physician trained in infectious diseases
Prion disease (1)	Case-based learning, clinical integration	Human medicine physician trained in infectious diseases
Human-animal bond (1)	Didactic, simulation, reflection	Academic topic expert and non-profit community expert
Veterinary perspective of climate change (2)	Didactic, case-based learning, clinical integration, reflection	Practicing veterinarian and academic topic expert
Visit to two local county departments of public health (1)	Field visit	Local public officials and experts
Visit to local county office of emergency management (2)	Field visit, role-play, and simulation	Local public officials and experts
Visit to JBS (local meat professing company, corporate office) to attend “Pork University” to understand the raising, processing, food safety measures, sustainability, and distribution of pork around the world (1)	Field visit, didactic	Private sector experts
Visit to JBS beef (local meat processing facility) to understand and experience the raising, processing, food safety measures, and distribution of beef around the world (2)	Field visit, didactic, experiential learning, reflection	Private sector experts
Visit to Colorado State University Spur: a free educational destination in Denver with a focus on food, water, and health research and innovation (1)	Field visit, didactic, small-group discussion, experiential learning, critical appraisal, reflection	Academic experts
Colorado Division of Reclamation and Quarry Mine Visit: a collaborative visit with the local Department of Mining to a local working mine to highlight the links between environmental hazards to human and ecosystem health (2)	Field Visit, didactic, small-group discussion, experiential learning, reflection	Private sector

(1), Occurred in Immersion 1. (2), Occurred in Immersion 2,

**TABLE 4:** Independent, asynchronous learning activities.

Learning activity	Format
Book club - *Zoobiquity* by Barbara Natterson-Horowitz and Kathryn Bowers	Critical appraisal, reflection, small-group discussion in Immersion 2 following independent work done during asynchronous period, peer-based learning
Journal club – students each chose one article to present. see references for full citations (18–23)	Critical appraisal, reflection, small-group discussion in Immersion 2 following independent work done during asynchronous period, peer-based learning
SEAOHN One Health modules: https://www.seaohun.org/one-health-modules	Didactics
Attendance at any five local inter-disciplinary One Health events of the students’ choice. An extensive list of possibilities are given to students and students are also allowed to add other events to this list as they learn about them.	Community engagement, experiential learning, reflection

### 3.4 Capstone project

The course culminated in a capstone project in which each student was asked to select a specific One Health challenge in a particular population and to create a framework for solving that One Health challenge. Students chose their topic during Immersion 1 and did the majority of their research work and presentation build during the asynchronous portion of the course. Students had the opportunity to connect with any of the interdisciplinary experts involved with the course or to reach out to other experts on their own. The course directors were involved in assisting students with topic selection, their approach to their topic, and final slide review. Students presented this work at an interdisciplinary conference made up of experts in the fields of human medicine, veterinary medicine, and environmental health. Interprofessional discussion with experts from various fields, including the deans from both the medical and veterinary schools and the director of the One Health Institute, provided valuable insights and an opportunity for a unique multidisciplinary approach that is not common in medical schools. Students were given feedback on their work and were given ideas for further opportunities in which they could present, disseminate, or build upon their work.

## 4 Results

To date, a total of 23 students from the Classes of 2025 and 2026 have participated in multiple different course and immersion evaluations. Among the Class of 2025, 8 students evaluated Immersion 1, and 5 students evaluated Immersion 2. From the Class of 2026, 4 students evaluated Immersion 1, and 6 students evaluated Immersion 2. Following each immersion, all students from both cohorts rated the curriculum on a six-point Likert scale. It is important to note that between the immersions, some students choose to take a year away from the traditional medical school curriculum to pursue research or another advanced degree. A total of 11 students have completed both immersions. Thus, the same number of people do not participate in each immersion.

A critical component of the course involved experiential learning through visits to community-based sites. All students *strongly agreed* or *agreed* that the immersion site visits were effective for their learning, and many identified this as the most impactful part of the course. Additionally, 95.7% of students *strongly agreed* or *agreed* that they understood the role of public health departments in safeguarding community health. A total of 100% of students reported an improved understanding of the role of organizations such as the CDC in maintaining public health. After Immersion 2, all students (100%) reported increased understanding of the mining industry, its regulatory frameworks, and the interconnections between human, animal, and environmental health. Notably, following a visit to JBS, one of the world’s largest meat processing companies, all students demonstrated an improved understanding of the impact of food production and food safety on human health. One student wrote, “I felt that all the (experiential learning activities) were effective for learning because it helped us to gain community, environmental, and future patient perspectives. I feel this is a particular strength of this trail.”

Reviewing data from the didactic portion of this course, we found that, by the conclusion of Immersion 2, most students had positive experiences. For example, 100% of students in the Class of 2025 and 83.3% of students in the Class of 2026 *strongly agreed* or *agreed* that they had gained a clearer understanding of how to incorporate One Health principles into their future medical practice. Additionally, 81.7% of both cohorts reported feeling empowered to address community health challenges through a One Health lens. All students (100%) either *strongly agreed or agreed* that they felt more confident collaborating with public health professionals to address interdisciplinary health issues. Furthermore, 90% of students from both cohorts indicated that the course improved their understanding of local emergency response systems and health-related frameworks. All students (100%) reported a clear understanding of how human-animal bonds and water quality impact human health. In the Class of 2026, by the end of Immersion 2, 100% of students felt that they had improved understanding of how climate change impacts the ecosystem, as well as human and animal health. Furthermore, 100% of future physicians from the Class of 2026 recognized various methods to advocate for their communities whether it be at bedside, local or national levels. By the end of the course, 81.8% of both cohorts reported an increased ability to generate a clinical differential diagnosis informed by One Health concepts, and 90.9% indicated that the knowledge gained helped them integrate and apply content from other parts of the medical curriculum to improve patient care.

## 5 Discussion

The One Health Trail: Physician Leaders as Advocates for Human, Animal, and Environmental Health offers a practical and impactful approach to integrating the One Health framework into undergraduate medical education. In a world where the interconnectedness of human, animal, and environmental health is increasingly evident, it is imperative to train future physicians to approach patient care holistically. Regardless of specialty, physicians have a responsibility to advocate not only for individual patients but also for the broader systems that influence health.

Physicians are traditionally trained as experts in human biology, disease, and pharmacotherapy. However, understanding external factors, such as zoonotic disease transmission, environmental degradation, water and air quality, and food safety, is essential for comprehensive patient care. The One Health framework empowers medical students to broaden their clinical perspective and critically evaluate health determinants that extend beyond the walls of the hospital or clinic.

Student evaluation data suggests that students who participate in this course develop the skills necessary to identify and address complex health challenges through systems-level thinking. They are introduced to topics and community-based experiences typically encountered later in their careers, such as interprofessional collaboration, environmental health, and public health policy. It should be underscored that integrating One Health into a medical curriculum requires more than adding new content. Instead, it requires bringing together diverse experts who can help medical students see health issues in their full biological, environmental, and social complexity. This was done throughout the course in a variety of ways. For example, a simulated wildfire response exercise with the local department of emergency management, demonstrated to students the importance of multi-disciplinary collaboration to ensure the health of humans, animals, and the environment in the setting of a wildfire. Students come away with the reinforcement that the One Health lens is essential in disaster management. Another example was a visit to a local mine that was led by the actual mining company and workers. Interacting with mine operators and local regulation agency representatives expanded students understanding of the causes, contexts, and solutions of health issues related to mining. This allows a perspective shift from a purely biomedical problem or a disease in a patient to a risk in a system that involves humans, animals, and the environment. Within this framework, students learn to analyze how the health of patients, animals, and their shared environments interact to influence clinical outcomes.

Capstone projects highlight the application of One Health principles in diverse contexts. For example, one student explored food security through a One Health lens, analyzing the roles of various stakeholders in ensuring sustainable and equitable access to safe food and clean water for both people and animals. Another student examined cross-disciplinary collaboration between physicians and veterinarians—proposing ways to integrate techniques from adult and pediatric medicine, such as Advanced Trauma Life Support (ATLS), into veterinary practice, and vice versa. They noted, for instance, how insights from veterinary medicine, like the progression of joint disease in animals, could inform preventive strategies in human medicine. Students also learned how to provide empathetic, culturally sensitive care, particularly for Indigenous communities whose relationships with animals and the environment are central to their identity and well-being. These experiences helped students confront and understand the social injustices embedded in health systems and consider how One Health-oriented solutions might contribute to more equitable care.

From the perspective of course directors, key lessons have emerged from working with the first two cohorts. First, community-based experiences are essential. Both subjective and objective data show that students gain a deeper understanding of the social and environmental determinants of health through immersive, real-world engagement. These opportunities prepare them to advocate effectively for community-driven solutions. Second, teaching students to analyze health issues through a One Health lens encourages long-term, sustainable thinking. Solutions developed in this way often have broader and more lasting impacts across multiple generations.

Feedback from students was largely positive with one student commenting that they felt like all medical students should be required to take this course. A few students commented that the work load was larger than what they had expected and that engagement in the community sometimes lead to long drives that were time-consuming. That being said, students consistently preferred experiential learning over traditional classroom didactics. These community-engaged, interdisciplinary approaches are not only more memorable but are likely to have a lasting influence on students’ clinical practice and advocacy efforts as they enter the healthcare workforce. As course directors, we advocate that the content addressed in this novel curriculum be adapted and incorporated into all U.S. medical schools.

## 6 Acknowledgment of constraints

This curriculum and its associated data collection have limitations. Most notably, the current evaluation is based on a small cohort of students, which limits the generalizability of findings. There is also a potential selection bias, as students who choose to take this course may already have an inherent interest in One Health concepts. Additionally, while the course content was designed to be broadly applicable to medical practice across diverse settings, some elements are inherently region-specific. For example, collaboration with the local public health department on wildfire emergency preparedness is highly relevant in Colorado but may be less applicable in regions such as Florida. Nevertheless, the underlying competencies—such as disaster preparedness and interagency collaboration—are transferable and can be adapted to other environmental contexts. Other medical schools seeking to integrate this curriculum at their campus could adopt content applicable to all learners regardless of geographic location, such as pandemic prevention, human-animal bond, or climate change, and then also work with their own local policymakers, non-profits, academic experts, and community-based organizations to integrate local, community-specific One Health challenges to the course. In this way, the course is highly adaptable across different institutions for undergraduate medical education.

As the program continues, it is expected that larger student cohorts and ongoing evaluation will strengthen the data set and inform iterative improvements to the curriculum. Despite these limitations, the One Health Trail represents a valuable and adaptable model for integrating One Health principles into medical education. With minor modifications to reflect local environmental and public health priorities, this curriculum could be implemented effectively at other medical schools, both nationally and internationally.

## Data Availability

The raw data supporting the conclusions of this article will be made available by the authors, without undue reservation.
